# Population Behavior Regarding the Use of Face Masks to Prevent the Transmission of Respiratory Infections: Lessons to Be Learned from the COVID-19 Pandemic

**DOI:** 10.3390/ijerph22020147

**Published:** 2025-01-22

**Authors:** Lucia Ingridy Thorpe, Jefferson Renato Silverio da Silva, Simone Maria Muniz da Silva Bezerra, Marilia Perrelli Valença, Danielle Christine Moura dos Santos, Regina Celia de Oliveira, Fábia Maria de Lima, Claudia Santos Martiniano Sousa, Aurelio Molina da Costa, Rosilane de Lima Brito Magalhães, Isabel Cristina Ramos Vieira Santos

**Affiliations:** 1 Associate Postgraduate Program in Nursing, Nursing School, Saint Amaro Campus, University of Pernambuco and State of Paraíba, Recife 50100-130, Brazilfabia.lima@upe.br (F.M.d.L.);; 2 Life Sciences Center, Nursing School, Catholic University of Pernambuco, Recife 50050-900, Brazil; 3 Postgraduate Program in Nursing, Nursing School, University of Piauí, Recife 64049-550, Brazil

**Keywords:** communicable diseases, prevention of diseases, face masks, COVID-19

## Abstract

Historically, the use of a face mask has been associated with personal protection during epidemics. However, the type of mask used and the way it is used can affect the level of protection it provides. To analyze the practices of using face masks in the population in the Northeast of Brazil, a cross-sectional study was carried out, from December 2021 to February 2022, through interviews with 308 people at bus stops (simple random sample). Pearson’s Chi-square test was calculated to verify the association between exposure and outcome variables. The prevalence of clinical manifestations of COVID-19 was 21.4%. The most used type of mask was made of one- and two-layer fabric when compared to N-95 and a three-layer surgical mask and its use were associated with people in the age group of 18–39 years, an income less than four minimum wage and education level equivalent to elementary/secondary school. An association was found between the infrequent use of a mask in a public environment, removing or lowering the mask when coughing and not washing hands before removing the mask and the occurrence of clinical manifestations suggestive of COVID-19. This study showed that socioeconomic factors are associated with the type of face mask used by the population and that the hygiene behavior of face-mask users was also associated with the occurrence of clinical manifestations of COVID-19. This highlights the need for guidelines and educational strategies that address these aspects to better protect the population against possible respiratory epidemics, especially in countries with important risk factors related to the use of face masks, and highlights the need for clear and objective guidelines and educational strategies to better protect the population against possible epidemics.

## 1. Introduction

During the COVID-19 pandemic, the high transmissibility of the virus, combined with the population’s lack of prior immunity, contributed to an exponential growth in the number of cases and motivated the adoption of preventive measures at individual, environmental and community levels, such as: hand hygiene, respiratory etiquette when coughing or sneezing, and the use of a face mask [1,2,3,4].

Historically, the use of a face mask has been associated with individual protection since the bubonic plague, when masks shaped like a bird’s beak were used to neutralize the “miasma” considered to be the cause of the disease [5].

These devices are designed to protect both the person using them and the immediate environment from bacterial or viral pathogens. The different masks can be classified as: complete, which covers the entire face; half mask, when it extends from under the chin to above the nose and, a quarter mask, referring to those that fit from the top of the nose to the top of the chin [6].

Following the declaration of a public health emergency, widespread use of masks outdoors led to a shortage of these devices in the market [7,8], compromising the ability of health systems in many countries to provide care to sick people. Therefore, the World Health Organization recommended the manufacture of cloth masks and their use in the community [7].

Although experimental evidence demonstrates that masks represent effective physical barriers to prevent the transmission of respiratory diseases, there is little empirical research on the use and effectiveness of masks to prevent the transmission of respiratory diseases in community settings. Furthermore, not only is the material used to manufacture face masks important, but other factors may also interfere with the protection provided by the mask, such as the user’s behavior regarding handling and hygiene practices [6,9,10,11].

Therefore, this study aimed to analyze the behavior of face-mask users to prevent the transmission of a respiratory infection.

## 2. Materials and Methods

### 2.1. Participants and Data Collection

Data collection was carried out from December 2021 to February 2022, at bus stops on the main avenues of the six regions of a city in northeastern Brazil, so that the sample would be representative since at bus stops it is possible to find people who represent the largest portion of the population, with regard to age, sex, and socioeconomic conditions.

The choice to collect data at bus stops was justified by the large flow of people and high exposure to COVID-19. Considering the estimated population of 1.555 million people and the proportions of 31.3% women and 26.9% men, in the age group of 18 to 60 years, a simple random sample was calculated, estimated at 308 people.

The statistical parameters: Confidence level of 95%, margin of error of 5%, and frequency expected 50% were considered for the sample calculation.

The eligibility criteria used were: people of both gender, aged between 18 and 60 years, with self-reported cognitive ability to answer the questionnaire. In addition, people who were wearing a mask partially covering their nose and/or mouth and those who had already been hospitalized due to COVID-19 were excluded.

### 2.2. Survey Variables

Data were collected through a questionnaire on behavior regarding the use of face masks to prevent the transmission of respiratory infections, developed by the researchers (content validation by scale level = 0.90; and Cronbach’s alpha = 0.85) and applied after explaining the research objectives and obtaining the participant’s consent through the signing of the free and informed consent form. The questionnaire consisted of 21 questions regarding socioeconomic variables, type of mask, behavior regarding its use, and clinical manifestations of COVID-19. For analysis, the case definition proposed by the WHO was used: presence of symptoms such as cough, sore throat or runny nose, anosmia, ageusia, diarrhea, abdominal pain, fever, chills, myalgia, fatigue, and/or headache [12].

### 2.3. Statistical Analysis

The data were analyzed using descriptive statistics, using mean (X) and standard deviation (SD). A Pearson’s Chi-square test was calculated to verify the association between exposure and outcome variables, considering a *p* value of 0.05 for statistical decisions. The analysis was carried out using the statistical package for social sciences—SPSS version 23.

### 2.4. Ethics Statement

The following information was provided to participants: participation in the study was voluntary; there would be no identification of the names of the participants; consent to participate in the study must be given by signing the free and informed consent form; there would be no cost to participate in the study. This study was conducted in accordance with the Declaration of Helsinki and approved by the Ethics Committee of the Oswaldo Cruz University Hospital (Pernambuco, Brazil; approval no. 5083046).

## 3. Results

### 3.1. Basic Characteristics of the Participants

The sample was characterized by an age range of 18 to 39 years (Average: 30.4; SD: 9.6), with an income greater than or equal to four minimum wages (Average: US$ 480.10; SD: US$ 144.03). The higher proportion of participants had completed secondary education and declared that they had no religion. Regarding the type of mask, a higher frequency of the two-layer cloth mask was observed (Table 1).

### 3.2. Behavior of the Population Regarding the Use of Face Masks

Table 2 presents the behavior of the sample studied regarding the use of masks. Despite the greater proportion of people interviewed reporting that they always wear a mask in a public environment, the significant proportion of those who do not do so can be noted, which together with those who rarely use it, reaches a percentage of 25%. Just over half of the sample reported that they frequently adjust the mask during use. A percentage of 42.2% lower or remove the mask to cough, and 78.6% of people do not wash their hands when removing the mask.

Table 3 presents the results regarding the existence of an association between the type of face mask used and sociodemographic variables. An association was observed between age and the type of mask used by the sample, in which the group corresponding to 18 and 39 years old most frequently used a one- or two-layer cloth mask, while those aged between 40 and 60 years old opted for the use of N-95 and surgical masks.

People’s income is also associated with the type of mask used: people with lower incomes used fabric masks with a statistically significant difference compared to people with higher incomes.

With regard to education, the largest proportion of people who were in elementary or secondary education used cloth masks, with a statistically significant difference.

The only variable analyzed that did not show an association with the type of mask was religion, despite the higher proportions of those using cloth masks, except for the spiritist religion and others.

The prevalence of clinical manifestations of COVID-19 in the studied sample was 21.4% and according to the results presented in Table 4, there was no statistical association between the types of mask used. However, an association was observed between the occasional use of a mask, the behavior of removing or lowering the mask to cough and not washing hands before removing the mask, and the occurrence of clinical manifestations of COVID-19.

## 4. Discussion

It has been shown that measures such as physical distancing and early vaccination have contributed significantly to reducing the level of infection in the environment, reducing the likelihood of becoming infected [13]. However, in the case of the COVID-19 pandemic, the use of face masks was a prophylactic measure prior to vaccination.

The use of face masks by the population as a measure to prevent respiratory infections continues to be the subject of great debate, whether due to the relationship between supply and demand, as occurred during the COVID-19 pandemic, or mainly due to knowledge gaps regarding their effectiveness.

In this study, it was observed that the most used type of mask was the double-layer fabric and its use was associated with the young age group, income below four minimum wages, and education corresponding to elementary or secondary education.

Regarding the type of mask, some authors claim that masks made of fabric have reduced effectiveness when compared to N95 and surgical masks; however, when manufactured with a double layer, their effectiveness can be increased [11]. It is estimated that creating masks with multiple layers of fabric or with a higher thread count improves filtration; however, their filtration capacity is also related to the design and fit to the user’s face. Therefore, a standard for fabric masks is needed to assist users when purchasing or making them [14,15].

Although the literature on the association between socioeconomic variables and the behavior of their users is scarce, it is easy to understand the results found in this study regarding the use of cloth masks and age, income, and education. Especially with regard to income, the cost of an N95 mask is much higher than the cost of a mask made by the user himself with materials often available in his own home. Our results agree with a study conducted in the United States that highlighted the use of face masks by respondents with a lower income [16].

Furthermore, since there is little regulation on the reuse of face masks, the prevailing wisdom is that cloth masks have greater strength and durability compared to N95 and surgical masks, making them cheaper and more affordable for people with limited incomes.

Unlike the findings of Rader et al. [16], the association found between the use of cloth masks and the age group of 18–39 years can be justified primarily because people in this age group correspond to 37.9% of the Brazilian population, are economically more active, and are more exposed to public environments than the elderly. Likewise, low education levels may be related to the high percentage of people in this age group who had not completed elementary schooling in the Brazilian population, determining the lack of knowledge about the effectiveness of the protection provided according to the type of mask.

Although there was no association between the type of mask used and religion, some studies claim that despite the general public’s adherence to the use of face masks, there are some groups in society who refuse to use them, based on religion, social class, and ethnicity [17,18].

There was also no association between the type of mask used and the occurrence of clinical manifestations of COVID-19, which initially suggests that there is some protection provided by fabric masks, especially those with two layers. This corroborates the results of studies that tested multilayer fabric masks in the laboratory, demonstrating that they have a blocking capacity of 50 to 70% for small droplets and exhaled particles [14,19,20].

Furthermore, not only is the use of a face mask important, but also the way in which the population uses it, in addition to its combination with other protective behaviors, such as social distancing and correct and frequent hand hygiene, which certainly contributes to the prevention of respiratory infections, reducing the speed of dissemination and, consequently, the number of cases and deaths [8].

The results found here demonstrate the existence of an association between the occurrence of clinical manifestations of COVID-19 and the occasional use of a face mask, the behavior of removing the mask to cough and not washing hands when removing the mask.

The mandatory use of face masks covering the nose and mouth has been determined by Federal regulation in Brazil since July 2020, applying to the movement of people in public and private environments, on public roads, and on public transport [21]. However, this law does not provide for punitive actions for offenders. Occasional use may be related to complex factors such as the socioeconomic and cultural vulnerability of the population and unfavorable climatic conditions with high temperatures typical of tropical countries [8,15].

As already demonstrated in the literature, the virus is transmitted mainly through exhaled respiratory droplets and its concentration can increase considerably when infected people remain for prolonged periods in environments with limited air exchange, facilitating transmission [15]. The occasional use of a face mask both leaves the user exposed to contamination and facilitates the spread of the virus by infected people, even if asymptomatic.

Virus transmission occurs through exposure to potentially infectious respiratory droplets spread by coughing and sneezing. Exposure increases directly the closer the distance from the source is and according to the rate and strength of airflow during exhalation [7,15]. In this sense, the results of a study that verified the effect of mask use on cough airflow demonstrated that the mask impedes the natural mechanism of airborne transmission of the infection, either by blocking the formation of the jet or by redirecting it in a less harmful direction [10].

Both international institutions and public health representatives in Brazil recommend respiratory etiquette as part of protective measures, which emphasize the practice of covering the nose and mouth when coughing and sneezing, as well as avoiding touching the eyes, nose and mouth with unwashed hands [22,23], preventing viruses from being easily transmitted to other people or to the environment.

The results found in this study show that the behavior of removing or lowering the mask to cough constitutes a risk situation and demonstrates the cultural fragility that some populations may present in relation to good respiratory etiquette practices and reinforces the need for greater commitment to educational strategies, on the part of public management and non-governmental institutions interested in controlling respiratory infections in a pandemic situation.

This study also observes an association between the behavior of not washing hands before removing the mask and the occurrence of clinical manifestations of COVID-19. This corroborates the need for greater clarity in educational strategies for the population regarding the practice of hand washing, both when putting on and taking off the mask, to avoid self-contamination through the possible touching of surfaces, objects, and people.

Research on mask-wearing behavior highlights the need for effective community education on the correct way to wear face masks and hygiene practices associated with their use.

This study presents as strengths the associations between the type of face mask used by the population and socioeconomic variables, and between manifestations of COVID-19 and factors related to the hygiene behavior of face-mask users, unprecedented results that can contribute to measures to prevent other respiratory epidemics. However, it presents limitations attributed to the study design, such as: it does not allow cause and effect relationships and those related to the veracity of the answers provided. These limitations must be taken into account when interpreting the results presented.

## 5. Conclusions

The results show that not only adherence to the use of face masks is important to prevent the transmission of respiratory infections, but also aspects related to the type of manufacture and the hygiene behavior of the population that uses them. The fact that no association was found between the occurrence of clinical manifestations of COVID-19 and the type of face mask used, in principle, indicates that fabric masks can provide some protection to users.

The discovery of an association between clinical manifestations of COVID-19 and the occasional use of a face mask in public settings, the behavior of lowering or removing the mask when coughing, and not washing hands before removing it, provides evidence on other important aspects for preventing the transmission of respiratory infections, which should be part of public health guidelines and activities, especially in low-income countries.

## Figures and Tables

**Table 1:** Characterization regarding sociodemographic variables and type of mask used.

Variable	*n* (%)
Age of the participants (y)	
18–39	265 (86.0)
40–60	43 (14.0)
Income	
≤1 MW	69 (22.4)
1–2 MW	56 (18.2)
2–4 MW	67 (21.8)
≥4 MW	116 (37.7)
Education level	
Elementary	24 (7.8)
Secondary	153 (49.7)
Postsecondary	131 (42.5)
Religion	
Catholic	76 (24.7)
Protestant	83 (26.9)
Spiritist	28 (9.1)
Without religion	121 (39.3)
Type of face mask used	
N95	41(13.3)
Surgical mask	77(25.0)
1 layer cloth mask	71(23.1)
2 layer cloth mask	119 (38.6)

The data are presented as the *n* (%). MW, minimum wage (US $208.05).

**Table 2:** Behavior of face-mask users.

Variable	*n* (%)
Use in public environment	
Always	231 (75.0)
Often	67 (21.8)
Rarely	10 (3.2)
Mask adjustment	
Frequently	156 (50.6)
Rarely	152 (49.4)
Behavior during coughing	
Lower/remove the mask	130 (42.2)
Keep the mask on face	178 (57.8)
Wash hands before removing the mask	
Yes	66 (21.4)
No	242 (78.6)

**Table 3:** Association between the type of mask used and sociodemographic variables.

Variable	N-95/Surgical Mask	Cloth Mask 1 or 2 Layers	*p*
Age of the participants (y)			
18–39	94 (35.5)	171 (64.5)	0.010
40–60	24 (55.8)	19 (44.2)	
Income			
<4 MW	79 (41.1)	113 (58.9)	0.010
≥4 MW	39 (33.6)	77 (66.4)	
Education level			
Elementary/secondary	54 (30.5)	123 (69.5)	0.001
Postsecondary	64 (48.9)	67 (51.1)	
Religion			
Catholic	29 (38.2)	47 (61.8)	0.228
Protestant	26 (31.3)	57 (68.7)	
Spiritist	18 (51.4)	17 (48.6)	
Without religion	45 (39.5)	69 (60.5)	

y = years old. MW = minimum wage (US $208.05).

**Table 4:** Association between clinical manifestations of COVID-19, type of mask and behavior of face-mask users.

Variable	COVID-19		*p*
	Yes	No	
Mask type			
N95 e Surgical	23 (19.5)	95 (80.5)	0.077
1 layer cloth mask	22 (31.0)	49 (69.0)	
2 layer cloth mask	21 (17.6)	98 (82.4)	
Use in public environment			
Always	43 (18.6)	188 (81.4)	0.037
Sometimes	23 (29.9)	54 (70.1)	
Mask adjustment			
Frequently	40 (25.6)	116 (74.4)	0.068
Rarely	26 (17.1)	126 (82.9)	
Behavior during coughing			
Lower/remove the mask	49 (38.0)	81(62.0)	0.001
Keep the mask on face	17 (10.0)	161(90.0)	
Wash hands before removing the mask			
Yes	7 (10.6)	59 (89.4)	0.016
No	59 (24.4)	183 (75.6)	

## Data Availability

All relevant data are included in this article.
